# Metaplastic carcinoma of the breast and *BRCA1* germline mutation: a case report and review

**DOI:** 10.1186/s13053-020-00162-x

**Published:** 2021-01-06

**Authors:** Michiko Yamashita, Yoshiaki Kamei, Akari Murakami, Erina Ozaki, Kumiko Okujima, Kana Takemoto, Megumi Takaoka, Daiki Tsukamoto, Erina Kusakabe, Tomoyuki Shidahara, Haruna Noda, Reina Aoki, Kana Taguchi, Kanako Nishiyama, Mariko Eguchi, Yasutsugu Takada

**Affiliations:** 1 Department of Breast Center, Ehime University Hospital, Shitsukawa, Toon, Ehime Japan; 2 Department of Hepato-Biliary-Pancreatic Surgery and Breast Surgery, Ehime University Graduate School of Medicine, Shitsukawa, Toon, Ehime Japan; 3 Department of Total Medical Support Center, Ehime University Hospital, Shitsukawa, Toon, Ehime Japan; 4 Department of Pediatrics, Ehime University Graduate School of Medicine, Shitsukawa, Toon, Ehime Japan

**Keywords:** Chondroid metaplasia, *BRCA1* mutation, Triple-negative breast cancer

## Abstract

**Background:**

Metaplastic carcinoma of the breast consists of both invasive ductal carcinoma and metaplastic carcinoma. This rare subtype of cancer has a poor prognosis. The development of metaplastic breast cancer and relationship with *BRCA1* are not well known. Here, we report a rare case of germline *BRCA1* mutation-positive breast cancer with chondroid metaplasia.

**Case presentation:**

A 39-year-old Japanese woman with a family history of breast cancer in her mother and ovarian cancer in her maternal grandmother consulted at our hospital with a left breast mass. Needle biopsy for the mass was performed, leading to a diagnosis of invasive breast cancer with chondroid metaplasia. We performed left mastectomy + sentinel lymph node biopsy + tissue expander insertion and replaced with a silicone implant later. Pathological examination revealed that the patient had triple-negative breast cancer. Four courses of doxorubicin+ cyclophosphamide therapy were performed as adjuvant therapy after surgery. We performed genetic counseling and genetic testing, and the results suggested the germline *BRCA1* mutation 307 T> A (L63*). She has currently lived without a relapse for 2 years post-surgery.

**Conclusions:**

There have been only 6 cases of metaplastic breast carcinoma with germline *BRCA1* mutations including our case. Patients with *BRCA1* mutations may develop basal-like subtypes or M type of triple-negative breast cancer besides metaplastic breast cancers.

## Background

Most germline *BRCA* mutation-positive breast cancers are invasive ductal carcinoma of the non-special type; the frequency of special types, such as metaplastic carcinoma, is low. Here, we report a rare case of g*BRCA1* mutation-positive breast cancer with chondroid metaplasia and review the literature on g*BRCA1* mutation-positive metaplastic carcinoma of the breast.

## Case presentation

A 39-year-old woman with a family history of breast cancer in her mother and ovarian cancer in her maternal grandmother noticed a lump in her left breast and consulted her primary care doctor. Because of the examination, the possibility of breast cancer was considered, and she was referred to our hospital for detailed examination.

A 1.4-cm tumor was palpated in the upper-outer region of left breast, an enlarged lymph node was not palpated, and there was no nipple discharge.

**Fig. 1:**
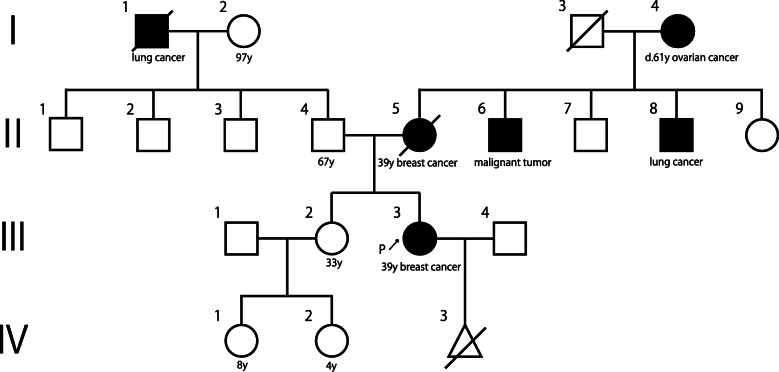
Family pedigree. The family history related to hereditary breast cancer and ovarian cancer syndrome includes breast cancer in the mother and ovarian cancer in her maternal grandmother

**Fig. 2:**
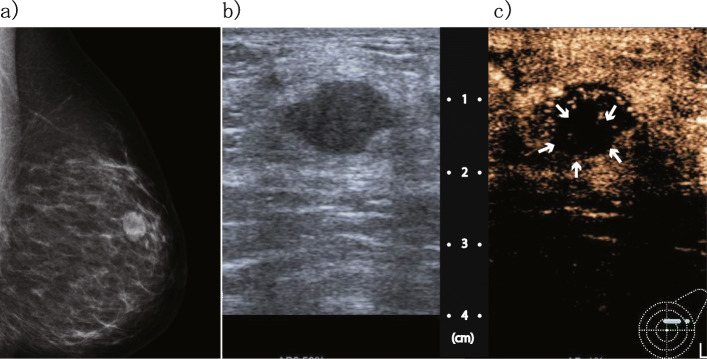
Imaging results. **a** Mammography; an equal density mass with calcification. **b** Ultrasound; a 1.4-cm microlobulated heterogeneous mass. **c** Contrast-enhanced ultrasound; arrows indicate contrast loss area

According to mammography (Fig. 1), an equal density mass with a margin showing a circumscribed irregular shape was observed in the outer upper quadrant of the left breast at one o’clock in the anterior portion, and calcification was found inside of the mass. By ultrasonography, a 1.4 × 1.4 × 1.0-cm clear and rough, microlobulated marginal heterogeneous mass was observed in the outer upper quadrant of the left breast, along with calcification and enhanced posterior features (Fig. 2b).

Invasive ductal carcinoma and adjacent chondroid metaplastic breast cancer were diagnosed by needle biopsy. Positron emission spectroscopy-computed tomography revealed a tumor of maximum standardized uptake value of 7.7 in the outer upper quadrant of the left breast, with no lymph node metastasis or distant metastasis.

Based on these results, we diagnosed the patient with left breast cancer, cT1cN0M0, Stage I.

We performed left mastectomy + sentinel lymph node biopsy + tissue expander insertion as well as silicone implant replacement surgery approximately 6 months later.

**Fig. 3:**
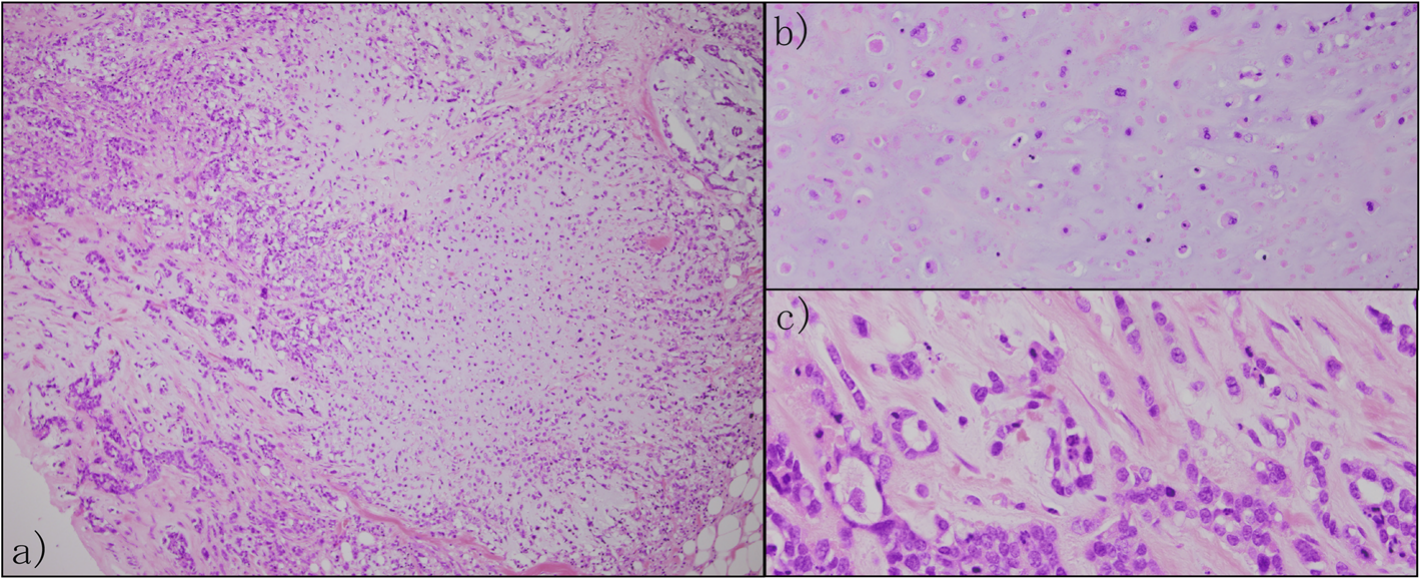
Pathological findings. **a** Low-magnification image (× 10); bright part on the right area is a metaplastic carcinoma component containing cartilage matrix, and the part with a large number of nuclei on the left area is a ductal carcinoma component. **b** High-magnification image: metaplastic carcinoma lesion (× 20); component of metaplastic carcinoma, type of chondroid metaplasia. **c** High-magnification image: ductal carcinoma lesion(× 40); component of ductal carcinoma, strong atypia, and mitosis in the nuclei

In the outer upper quadrant of the whole breast resection specimen, 1.5 cm of metaplastic carcinoma (Fig. 3a), a component of chondroid metaplasia (Fig. 3b), and a component of ductal carcinoma (Fig. 3c) were mixed. Breast cancer invaded the fat tissue, the nuclear grade was 3 (atypia: 3, mitosis: 3), and the surgical margin was negative. Lymph node metastasis was not observed. The results of immunohistochemistry analysis were as follows: estrogen receptor: negative (0%), progesterone receptor: negative (0%), HER-2: negative (0), and MIB-1 index: 70–80%. These results suggested triple-negative breast cancer (TNBC).

As a postoperative course, we conducted genetic counseling because of the young onset of TNBC with a family history of both breast cancer and ovarian cancer. She wished to undergo genetic testing, and thus we evaluated *BRCA1* and *BRCA2*. The results confirmed the presence of the pathogenic mutation c. 188 T>A (L63*) in *BRCA1*. Four courses of doxorubicin+ cyclophosphamide therapy were performed as postoperative adjuvant chemotherapy. In addition, after disclosing the genetic test results, periodic surveillance of ovarian cancer was started. She did not want to undergo contralateral mastectomy; therefore, surveillance of the contralateral breast was started. The patient has asthma and cannot be evaluated by contrast-enhanced magnetic resonance imaging, and thus mammography and ultrasonography are performed once per year. Currently, she has been alive without relapse for 2 years post-surgery.

## Discussion and conclusions

Metaplastic carcinoma includes a group of tumors in which adenocarcinoma has caused “metaplasia” to differentiate into the squamous epithelium, mesenchymal component, spindle cell, cartilage, bone, and others [1].

The frequency of metaplastic carcinoma in breast cancer is reported to be approximately 1%, making this condition quite rare [1]. Almost all metaplastic breast cancers are classified as TNBC [2].

Approximately 80–90% of breast cancers developed in carriers with pathological mutations in *gBRCA1* are classified as the basal-like subtype in the gene expression profile, most of which show characteristics of TNBC [3]. Based on this, Perou et al. reported that *BRCA1* is frequently observed in different stages of epithelial cell development; a *BRCA1* mutation is linked to this luminal progenitor/basal-like phenotype, and loss of *BRCA1* may block further differentiation and retain a cell in this stage of development [4].

Our case and only 5 other cases have been reported according to PubMed, showing that a g*BRCA1* mutation-positive status may lead to metaplastic cancer development [5–8].

All six cases were TNBC; however, the *BRCA1* mutation site and tissue type of metaplastic carcinoma differed (Table 1). In previous reports, g*BRCA1* mutation-positive metaplastic carcinomas included carcinosarcoma in 2 cases, osseous and chondroid metaplasia in 1 cases, squamous cell carcinoma in 1 case, and adenosquamous carcinoma in 1 case. A case of osseous and chondroid metaplasia was examined for breast cancer tissue at the Foundation One CDx and it is unclear whether it is a germ line mutation or a somatic mutation [9]. Another case of *BRCA1* mutation-positive metaplastic breast carcinoma was reported many years ago [10] but a search in the current database revealed that the mutations were not pathogenic.

**Table 1:** Reported cases

	Age	Family history	*BRCA1* mutation	Subtype	Pathological type
Rashid et al [5].	22	none	c.66_67 (E23fs)	TNBC	carcinosarcoma
Noel et al [6].	49	none	c.66dupA (E23fs)	TNBC	adenosquamous carcinoma
Suspitsin et al [7].	35	mother 52 years ovarian cancer	c.5266dupC (Q1756fs)	TNBC	carcinosarcoma
Breuer et al [8].	25	two paternal aunts 40 years or younger breast cancer	c.181 T>G (C61G)	TNBC	squamous cell carcinoma
Hamad et al [9].	54	none	(G511fs)	TNBC	osseous and chondroid metaplasia
our case	39	mother 39 years breast cancer, maternal grandmother 61 years ovarian cancer	c. 188 T>A (L63*)	TNBC	chondroid metaplasia

Five cases of metaplastic carcinoma of the breast with germline mutations in *BRCA1* were reported. There were 2 cases of carcinosarcoma, 1 cases of osseous and chondroid metaplasia, 1 case of squamous cell carcinoma, and 1 case of adenosquamous carcinoma

Recently, in subtype classification based on the gene expression profile, TNBC was divided into four subtypes: BL1, BL2, M, and LAR [11]. BL1 and BL2 are characterized by the expression of genes related to the cell cycle and DNA damage response and are cisplatin-sensitive in cell lines.

M is characterized by epithelial mesenchymal transition-related gene expression, the expression of genes involved in the growth factor pathway, and sensitivity to the PI3K/mTOR inhibitor NVP-BEZ235 and the abl/src inhibitor dasatinib. The LAR subtype is characterized by an androgen receptor signal and is sensitive to the AR antagonist bicalutamide.

In a previous report, among the 10 patients with *BRCA* mutation in the TNBC subtype of breast cancer, 3 cases were reported for BL1, 4 for BL2, and 3 for M [12]. In another report, among the 28 metaplastic cancers, 12 cases were reported for M, 1 for BL1, 2 for BL2, and the 4 for MSL (currently, MSL is reclassified as BL or M), and 9 for unstable/unknown. The results of these two studies suggested that TNBC with *BRCA* germline mutation and metaplastic breast cancer are the same subtype, BL1, BL2, or M type TNBC [13].

Patients with *BRCA1* mutations may develop basal-like subtypes or the M type of TNBC, in addition to metaplastic breast cancers. There are no reports of LAR type breast cancer in either g*BRCA1* mutation-positive breast cancer or metaplastic breast cancer, suggesting that the disease is another strain.

In addition, according to gene expression analysis of somatic cells from patients with metaplasia breast cancer, the BRCAness signature is found in 5/8 of squamous cell carcinoma cases, 2/8 of chondroid metaplasia cases, and 1/10 spindle cell carcinoma case.

The BRCAness signature differed depending on the type of metaplastic carcinoma.

Thus, g*BRCA1* mutations cause metaplastic breast carcinomas, particularly chondroid metaplastic carcinomas, carcinosarcoma, and squamous cell carcinomas.

Only 6 cases of g*BRCA1* mutation-positive metaplastic breast carcinoma have been reported; however, based on developmental hypotheses and the results of gene expression analysis, g*BRCA1* mutations may be found in patients with metaplastic carcinoma at rates at least as high as those in TNBC. To determine the relationship between metaplastic carcinoma of the breast and pathogenic mutations in *BRCA1*, investigation of additional cases is necessary.

## Data Availability

All data generated or analyzed during this study are included in this published article.

## Abbreviations

germline *BRCA*: g*BRCA*
TNBC: Triple-negative breast cancer

## References

[1] Lakhani SR, Ellis IO, Schnitt SJ, Tan PH, Van de Vijver MJ. WHO classification of tumours of the breast. 4th ed: International Agency for Research on Cancer: Lyon; 2012.

[2] Weigelt B, Ng CK, Shen R, Popova T, Schizas M, Natrajan R (2015). Metaplastic breast carcinomas display genomic and transcriptomic heterogeneity [corrected]. Mod Pathol.

[3] Sorlie T, Tibshirani R, Parker J, Hastie T, Marron JS, Nobel A (2003). Repeated observation of breast tumor subtypes in independent gene expression data sets. Proc Natl Acad Sci U S A.

[4] Perou CM (2010). Molecular stratification of triple-negative breast cancers. Oncologist..

[5] Rashid MU, Shah MA, Azhar R, Syed AA, Amin A, Hamann U (2011). A deleterious BRCA1 mutation in a young Pakistani woman with metaplastic breast carcinoma. Pathol Res Pract.

[6] Noel JC, Buxant F, Engohan-Aloghe C (2010). Low-grade adenosquamous carcinoma of the breast-a case report with a BRCA1 germline mutation. Pathol Res Pract.

[7] Suspitsin EN, Sokolenko AP, Voskresenskiy DA, Ivantsov AO, Shelehova KV, Klimashevskiy VF (2011). Mixed epithelial/mesenchymal metaplastic carcinoma (carcinosarcoma) of the breast in BRCA1 carrier. Breast Cancer.

[8] Breuer A, Kandel M, Fisseler-Eckhoff A, Sutter C, Schwaab E, Luck H (2007). BRCA1 germline mutation in a woman with metaplastic squamous cell breast cancer. Onkologie..

[9] Hamad L, Khoury T, Vona K, Nestico J, Opyrchal M, Salerno KE (2016). A case of metaplastic breast cancer with prolonged response to single agent liposomal doxorubicin. Cureus..

[10] Bellino R, Arisio R, D'Addato F, Attini R, Durando A, Danese S (2003). Metaplastic breast carcinoma: pathology and clinical outcome. Anticancer Res.

[11] Lehmann BD, Jovanovic B, Chen X, Estrada MV, Johnson KN, Shyr Y (2016). Refinement of triple-negative breast cancer molecular subtypes: implications for neoadjuvant chemotherapy selection. PLoS One.

[12] Echavarria I, Lopez-Tarruella S, Picornell A, Garcia-Saenz JA, Jerez Y, Hoadley K (2018). Pathological response in a triple-negative breast cancer cohort treated with neoadjuvant carboplatin and docetaxel according to Lehmann's refined classification. Clin Cancer Res.

[13] Weigelt B, Kreike B, Reis-Filho JS (2009). Metaplastic breast carcinomas are basal-like breast cancers: a genomic profiling analysis. Breast Cancer Res Treat.

